# From evidence-based medicine to digital twin technology for predicting ventricular tachycardia in ischaemic cardiomyopathy

**DOI:** 10.1098/rsif.2022.0317

**Published:** 2022-09-21

**Authors:** Anouk G. W. de Lepper, Carlijn M. A. Buck, Marcel van ‘t Veer, Wouter Huberts, Frans N. van de Vosse, Lukas R. C. Dekker

**Affiliations:** ^1^ Department of Cardiology, Catharina Hospital, Eindhoven, The Netherlands; ^2^ Department of Biomedical Engineering, Eindhoven University of Technology, Eindhoven, The Netherlands; ^3^ Department of Biomedical Engineering, CARIM School for Cardiovascular Diseases, Maastricht University, Maastricht, The Netherlands

**Keywords:** ventricular tachycardia prediction, digital twin, artificial intelligence, hybrid modelling, clinical decision support

## Abstract

Survivors of myocardial infarction are at risk of life-threatening ventricular tachycardias (VTs) later in their lives. Current guidelines for implantable cardioverter defibrillators (ICDs) implantation to prevent VT-related sudden cardiac death is solely based on symptoms and left ventricular ejection fraction. Catheter ablation of scar-related VTs is performed following ICD therapy, reducing VTs, painful shocks, anxiety, depression and worsening heart failure. We postulate that better prediction of the occurrence and circuit of VT, will improve identification of patients at risk for VT and boost preventive ablation, reducing mortality and morbidity. For this purpose, multiple time-evolving aspects of the underlying pathophysiology, including the anatomical substrate, triggers and modulators, should be part of VT prediction models. We envision digital twins as a solution combining clinical expertise with three prediction approaches: evidence-based medicine (clinical practice), data-driven models (data science) and mechanistic models (biomedical engineering). This paper aims to create a mutual understanding between experts in the different fields by providing a comprehensive description of the clinical problem and the three approaches in an understandable manner, leveraging future collaborations and technological innovations for clinical decision support. Moreover, it defines open challenges and gains for digital twin solutions and discusses the potential of hybrid modelling.

## 1. Background

Survivors of acute myocardial infarctions (MIs) are exposed to an increased risk of malignant arrhythmias, specifically sustained ventricular tachycardia (VT) or ventricular fibrillation. Post-MI VTs typically occur more than 8 years after this index event [1] and are associated with morbidity and mortality, including sudden cardiac death (SCD). To prevent SCD in patients with a high risk of these post MI-VTs, implantable cardioverter defibrillators (ICDs) are indicated. These ICDs provide therapy during VTs by either overpacing or defibrillation. Current guidelines are based on left ventricular ejection fraction (LVEF) and the New York Heart Association (NYHA) classification (a classification to quantify the extent of heart failure) as parameters for VT risk stratification and, thus, aim for primary prevention by implantation of an ICD [2]. However, LVEF is a poor risk stratification tool leaving high-risk patients unprotected and potentially low-risk patients exposed to the risk of unnecessary ICD implantation [3]. More specifically, ICD implementation will only prevent one SCD out of 15 implanted patients [4]. Moreover, ICD is not a cure for VT and exposes patients to negative effects like (inappropriate) shocks or ICD storms. Also, the occurrence of VT as well as ICD therapy is associated with anxiety and depression [5] as well as with worsening of heart failure [6]. Therefore, there is a clinical need for individual risk prediction tools that reduce over- and under-treatment in this very large patient population and allow for more tailored VT treatment in patients at high risk of VT, beyond current guidelines. Careful selection of patients at high risk for VT in an early stage, would allow for improved preventive treatment, medication and ICD implantation.

Ablation is an invasive therapy which creates scar tissue in the VT circuit to block the conductance of electric current waves leading to VTs. Although earlier studies showed that preventive VT ablation reduced overall VT occurrence and ICD incidence in this patient group [7–9], patient-specific modelling has the potential to further improve this therapeutic concept. Individualized prediction models can contribute to (patho)physiologically driven ablation strategies with phenotyping of the morphological and functional substrate [10].

We identify three approaches that are currently used to gain either insight in the pathophysiology or to predict the risk of VT in post-MI patients. The first approach, ***evidence-based medicine*** [11], is the common approach in clinical practice and mainly consists of the combination of clinical guidelines with diagnostic information, history taking and medical knowledge complemented with patient-specific clinical data. These population-based guidelines are built on large clinical trials and provide important information about most likely outcomes. However, not all relevant clinical information can be measured and inter-individual variability is not considered.

The second approach is ***data-driven models***, which are part of the data-science field, and which can reveal relationships in data without any predefined knowledge. Besides traditional statistical models, artificial intelligence (AI) models such as machine learning (ML) and deep learning (DL) are important data-driven models which are slowly entering the clinic. Both traditional statistical and ML models aim to learn from data, but differences can be attributed to the fact that ML algorithms are spared from most assumptions and/or hypotheses that are often made when applying traditional statistical methods. Especially in this age of big data, these AI models hold the promise to unravel new knowledge, combine multimodal data, and make predictions based on previously observed data. Nevertheless, they need a large amount of data to allow for generalization. Additionally, causality is often missing, leaving only correlational interpretation which hampers true understanding and explanation of the underlying (patho)physiology. Moreover, they can only provide answers for the scope they were trained for and thus fail at providing other solutions beyond their modelling goals.

The third approach is an engineering approach consisting of ***mechanistic models*** and provides information about the (patho)physiological state of the individual patient using accepted theories, such as (bio)physical laws. They can provide additional mechanistically relevant information such as myofibre stress which is impossible to measure non-invasively. Moreover, these models can be made patient-specific and used to predict and explain disease development and treatment outcome if the underlying mechanisms are known. These models enable true insights in the effects of different diseases and treatments. However, unfortunately the underlying mechanisms are often not known, and, as a result, these models are mainly applied in research settings.

**Figure 1.**
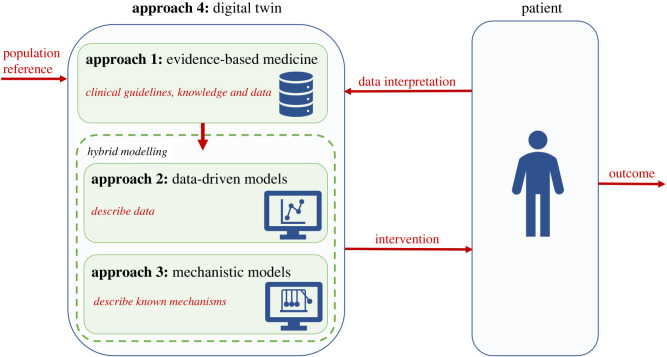
Digital twin concept. Schematic of a digital twin in which data-driven and mechanistic models are continuously being updated with clinical data from the patient. This hybrid model, in which data-driven and mechanistic models are combined, can be a decision support tool alongside evidence-based medicine for interventions decisions. This intervention affects the patients, thereby altering the data input. In this way, the digital twin is constantly evolving.

Currently, there is a shift in clinical practice from population to personalized clinical care, also known as precision medicine. We argue that precision medicine requires the integration of clinical data with mechanistic and data-driven models into a ‘***digital twin***' concept. A digital twin is a virtual model which integrates data-driven models and mechanistic models to represents an organ system or entire patient which is continuously updated with new clinical data [12]. The digital twin concept is schematically shown in figure 1. By combining data-driven models and mechanistic models, using hybrid modelling, evidence-based approaches can be complemented, which leads to an integrated approach which elevates the three independent approaches. So, hybrid modelling can be seen as a next generation approach introducing a new concept in current clinical decision support. However, designing such a concept is extremely challenging and requires a thorough understanding regarding the advantages and disadvantages of the three common approaches separately, as this will facilitate the design of new hybrid approaches. In addition, a strong collaboration between different domains, consisting of electrophysiologists, heart failure specialists, (bio)medical engineers and data scientists, is indispensable to fully exploit the possibilities of the different expertise. To achieve this, better mutual understanding of each other's points of view, knowledge, skills and limitations is essential.

Therefore, the aim of this paper is twofold. The first objective is to provide a comprehensive overview of the three currently available approaches and how these approaches are integrated and applied by the different expert fields. For this, the pathophysiological background of these VTs and the currently available clinical diagnostics supported with clinical studies are discussed first (§2). Further, an introduction to data-driven models and mechanistic models are given, including some state-of-the-art examples for VT and VT-ablation prediction (§§3 and 4, respectively). Not all studies will be discussed in detail, since there is an abundance of excellent clinical reviews [13,14], cardiac AI reviews [15–19] and cardiac mechanistic model reviews [20–23] available. The second objective builds on the insights obtained by the exploration of the existing literature and aims to provide our perspective on the open challenges and gains of Approach 4, the digital twin concept, and how it is envisioned to move VT prediction from population based to a patient-specific approach (§5). By linking clinical studies with state-of-the-art computer models, this review aims to bridge the first gaps between the various research domains required for such digital twin solutions.

## 2. Approach 1: evidence-based medicine

**Figure 2.**
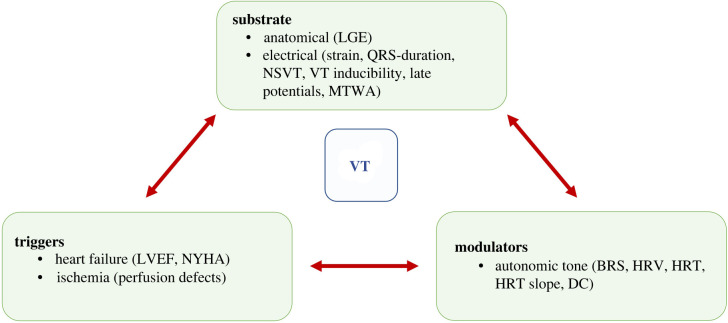
Phillipe Coumel's triangle. Figure of the interplay (using Coumel's triangle) of pathogenesis of ventricular tachycardia (VT). This figure provides an overview of the interplay between the substrate, triggers and modulators for VT to initiate and perpetuate. Between brackets the clinical measurements are indicated. LGE, late gadolinium enhancement; NSVT, non-sustained VT; MTWA, microvolt T-wave alternans; LVEF , left ventricular ejection fraction; NYHA, New York Hearts Association functional class; BRS, baroreflex sensitivity; HRV, heart rate variability; HRT, heart rate turbulence; DC, deceleration capacity.

There are three arrhythmogenic mechanisms: abnormal automaticity (accelerated, ectopic generation of an action potential by myocardial tissue), triggered activity (spontaneous depolarization occurring during or after repolarization) and re-entry [14]. In the ischaemic cardiomyopathy population, where patients have an oxygen deficit of the heart caused by reduced myocardial perfusion (such as MI) with related cardiac dysfunction, re-entry is the most important mechanism causing VT. To systematize the pathophysiology underlying re-entry, Philippe Coumel introduced the widely accepted triangle consisting of the substrate, the trigger and the modulator (figure 2). Where our review focuses on VT, several clinical trials that provide the basis for ICD usage use SCD as their endpoint. Even though this cannot be used interchangeably, the relevant studies will be used for reference in this section.

### 2.1. Substrate

A substrate is the pre-existing anatomical basis for the maintenance of an arrhythmia. Although somewhat artificial from a pathophysiological viewpoint, we subdivide this in anatomical and electrical components, as this most closely fits with commonly used diagnostic techniques supplying the data for prediction models.

**Figure 3.**
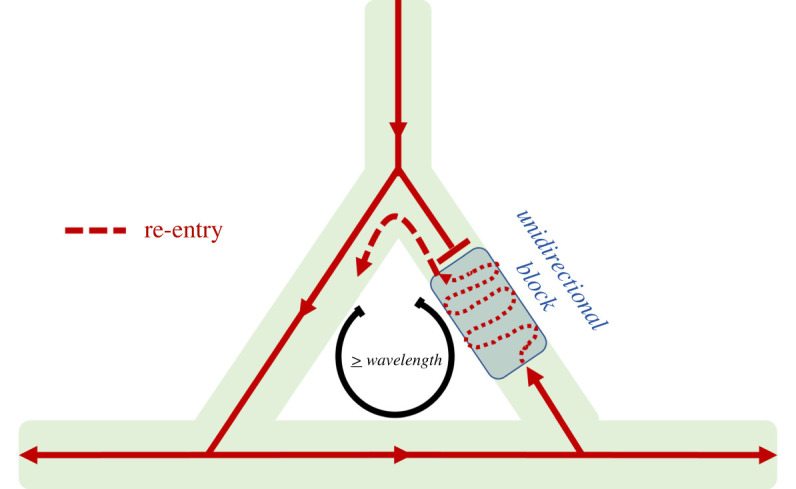
Schematic visualization of re-entry. A unidirectional block, after an extra-systole, prohibits conduction in antegrade direction in one limb. When the excitation wave reaches the other side, retrograde conduction occurs after recovery of the refractory period. In addition, within this infarcted area, conduction velocity is further decreased by the zig-zag pattern caused by disorganized myocardial fibre orientation.

For re-entry to be initiated and perpetuated, multiple conditions need to be met. Firstly, for initiation, a unidirectional block is required (figure 3). Secondly, for maintaining re-entry, the circuit length must be long enough to allow the myocardium in each part of the circuit to restore its excitability adequately (in other words, to pass its refractory period) to react to the next impulse [24]. Therefore, at least one wavelength, which is the product of conduction velocity and refractory period, must fit within the circuit. In infarcted tissue, intercellular coupling is altered and non-excitable regions exist. Here, the anatomical and electrical substrate are linked as conduction velocity is reduced, constituting another pro-arrhythmic condition. Also, several months after the MI, the anatomical orientations of the myocardial muscle bundles change. There is an increased complexity of the tracts with a more zig-zag conduction further increasing activation delay [24].

#### 2.1.1. Anatomical substrate

Fibrotic (i.e. scarred) strands, interdigitating with surviving myocardial fibres as well as gap-junction disarray create the anatomical arrhythmogenic substrate. Gap-junctions are cell-to-cell structures that allow ions to flow from one myocardial cell to the other. The fibrotic area consists of a border zone and a denser, infarct core. The border zone is considered the most important substrate for ventricular arrhythmias, since it consists of a mixture of scarred and surviving myocardium, leading to slow conduction which therefore promotes re-entry [25].

Cardiac magnetic resonance imaging (CMR), using late gadolinium enhancement (LGE), is the modality of choice to visualize scarring. Gadolinium is a contrast agent that penetrates extracellular tissue and therefore visualizes fibrotic tissue. This can picture the size of the peri-infarct border zone in relation to the infarct core. The size of the border zone is associated with arrhythmic risk. As shown by Yan *et al.* [26], the risk of death is significantly higher with a larger peri-infarct border zone and lower LVEF. The anatomical characteristics of the peri-infarct zone are not necessarily confined to the border of the infarct and can be seen in central locations. The CMR-GUIDE study is currently being conducted in Australia and Europe, randomizing patients with LVEF 36–50% on CMR-guided prophylactic ICD implantation. This would provide insight in the role of myocardial scarring as an independent risk factor for sudden arrhythmic death, irrespective of systolic left ventricular function.

Next to scar, contraction heterogeneity also predicts arrhythmic events. Contraction heterogeneity can be investigated by mechanical dispersion, examined by strain echocardiography. Mechanical dispersion is defined by the standard deviation of time to peak negative strain. This hypothesis was investigated by Haugaa *et al.* [27]. The optimal cut-off for mechanical dispersion was greater than 47 ms. Each 10 ms increase in mechanical dispersion contains a hazard ratio (HR) of 1.7 (95% CI 1.2–2.5, *p* < 0.01) in prediction of arrhythmic events, indicating that patients with a mechanical dispersion of greater than 47 ms have 1.7 times increased chance of arrhythmic events per 10 ms increase.

#### 2.1.2. Electrical substrate

In post-MI scar tissue, there is cell-to-cell uncoupling and disarray leading to inhomogeneous and slow conduction as well as to more regional differences in action potential duration [27] which causes the electrical substrate. A typical action potential first depolarizes the myocardium, which activates contraction (shown as the QRS complex on an ECG) and consequently repolarizes the myocardium back to the resting membrane potential (shown as the T-wave on an ECG). After repolarization, there is a refractory period during which cells are not excitable for another action potential. The action potential duration in the midmyocardial (middle) and endocardial (inner) layers is longer compared with epicardial (outer) layer, creating heterogeneity in repolarization. In scar tissue, the heterogeneity in action potential duration is further increased, contributing to arrhythmogenesis, as shown in heart failure patients [28].

The electrical conduction in the heart can be assessed using different (non-)invasive diagnostic techniques. The electrocardiogram (ECG) shows duration of the QRS-complex and intraventricular conductions delays. Several studies show conflicting evidence regarding the predictive value of the QRS duration for ventricular arrhythmias. MADIT II [4], CHARISMA [29] and COMPANION [30] did not reveal QRS duration as a significant predictor for VT. On the contrary, SCD-HeFT [31] showed an improved survival in ICD therapy with QRS duration more than 120 ms. Therefore, QRS duration does not seem to be a relevant predictor in VT, but rather reflects more severe ventricular dysfunction [14]. Fragmented QRS complexes, however, are a marker of depolarization abnormality. They represent a conduction delay with inhomogeneous activation caused by myocardial scar among others, with a sensitivity of 86%, specificity of 89% and negative predictive value of 93% [32].

Late and slow impulse conduction as a marker of scarred myocardium can be visualized using late potentials on the signal-averaged ECG (SAECG). This technique cancels noise and can therefore detect very low-amplitude signals at the end of the QRS-complex, and thus the presence of a substrate for re-entry [14]. Especially, this filtered QRS duration has been shown to predict VT [33]. The clinical significance, however, is low, as shown in the CHARISMA trial [29], where the QRS width on SAECG only has an HR of 1.04. Therefore, SAECG is rarely used for risk stratification [34].

Another possible predictor for haemodynamically significant VT is the appearance of non-sustained VT (NSVT), which by definition lasts less than 30 s, examined by remote cardiac rhythm monitoring. In the thrombolysis era, studies indicated that patients with more than 10 ventricular premature beats per hour had an increased risk of death [35,36], whereas more contemporary studies from Hohnloser *et al.* [37], CARISMA [38] and REFINE [39] have shown that NSVT is not a significant predictor of cardiac mortality.

Microvolt T-wave or repolarization alternans (MTWA) examined during exercise ECG is another diagnostic tool possibly linked to arrhythmogenic mechanisms, including altered calcium- and potassium-metabolism as well as cell-to-cell conduction disturbances causing repolarization heterogeneity. A positive MTWA includes a subtle, regular, beat-to-beat variation in the contour, amplitude or polarity of the T-wave of the surface ECG measured during an exercise protocol. MTWA cannot be measured in atrial fibrillation, frequent ventricular premature beats, and patients who are unable to increase their heart rate [3]. This technique is rarely used in practice for risk stratification, since the negative predictive value is high, but the positive predictive value is low [40]. Clinical evidence is contradictory. The ABCD trial [41] and REFINE study [39] showed that MTWA is a significant predictor of adverse cardiac events with a negative predictive value of 95% at 1 year. The MASTER [42] and CHARISMA trials [29], on the other hand, showed no role for MTWA in prediction of VT. All these studies were conducted in patients with reduced systolic left ventricular function based on ischaemic cardiomyopathy.

Another method of examining the electrical substrate is by performing an electrophysiological study (EPS) to invasively test for VT inducibility. EPS is currently not routinely incorporated in clinical risk stratification, since inducibility has a low positive predictive value, but high negative predictive value. Patients with a negative EPS have a very low risk for an arrhythmic event (SCD 3% at 2 years) [43], with a negative predictive value of 88% [44]. Adding EPS to the electrocardiographic non-invasive risk factors in patients with preserved LVEF described above results in a positive predictive value of only 22% and negative predictive value of 100% [45]. Currently, an Australian trial, PROTECT-ICD, is investigating the role of EPS early after MI to guide ICD implantation. The current European guidelines [46] indicate EPS after MI for patients with suspicion of VT based on symptoms, i.e. palpitations, presyncope and syncope. In patients with ischaemic cardiomyopathy with reduced LVEF, a positive EPS gives an indication for ICD which reduces mortality significantly [47].

### 2.2. Triggers

#### 2.2.1. Heart failure

A myocardial scar can lead to a depressed LVEF and heart failure. Symptoms are classified by the NYHA classification. In the current ICD guidelines, LVEF and NYHA classification are the only two variables included for risk stratification. In heart failure, neurohormonal adaptions occur, such as activation of renin-angiotensin-aldosterone system and activation of the sympathetic nervous system (see also §2.3.1) [28]. These are initially compensatory, but as heart failure advances, become maladaptive and proarrhythmic. With decreasing LVEF, the end diastolic pressure and volume in the left ventricle will increase, which causes stretch of the cardiomyocyte. Both acute and chronic stretch play a role in the pathogenesis of VT. Acutely, this causes activation of stretch-activated membrane channels which shortens the refractory period [28]. In chronic stretch, the effective refractory period and mean action potential duration shorten, constituting another pro-arrhythmic factor [28]. Changes in the action potential can cause triggered activity as the initiator of VT. Triggered activity can occur as early or delayed afterdepolarization depending on the timing of the depolarization in the action potential. In heart failure, inward potassium channels are downregulated, causing early afterdepolarization which serves as a trigger for ventricular arrhythmias. Delayed afterdepolarizations result from altered calcium handling, which can be stimulated by increased sympathetic activity [28]. Therefore, progressive heart failure with decreased LVEF and subsequently increased stretch are important predictors of malignant VT.

Clinical trials have consistently shown the relationship between a decrease in LVEF, progression of heart failure, and the increase in SCD and VT risk. LVEF is currently most often derived from echocardiography. The MASTER trial [42] shows that each decline of 1% in LVEF comes with 5% relative increase for VT. This is in correspondence with the 21% increase in sudden death with every 5% decrease in LVEF found in the VALIANT trial [48]. Two trials of major importance regarding LVEF are the MADIT II [4] and the SCD-HeFT trials [31]. They consistently show that an ICD provides a significant mortality risk reduction in patients with LVEF below 35%. These two trials have led to the class I, level of evidence A recommendation for ICD for primary prevention in ischaemic heart disease with LVEF less than 35% in NYHA II-III after three months of optimal medical therapy who are expected to survive at least 1 year in good functional status. As shown in the follow-up of the MADIT II [48] the survival benefit depends on the general clinical risk category consisting of multiple clinical risk factors like age and kidney failure. The high-risk group experienced no significant survival benefit from ICD implantation (HR 0.84). In high-risk groups, including patients with more advanced heart failure, there are competing, non-arrhythmogenic, risks for mortality from heart failure and non-cardiac causes, which is not influenced by ICD therapy [49]. This was similar in the SCD-HeFT [31], where no improved survival was seen in patients with NYHA III (HR 1.16), while NYHA II patients had a significant survival benefit (HR 0.54). Likewise, mainstream heart failure treatment including β-blockers [42], ACE-inhibitors and angiotensin receptor blockers significantly reduce VT [30]. These studies signify that risk stratification is necessary to differentiate patients with ischaemic cardiomyopathy who are at risk for arrhythmic death from patients who have a high mortality risk due to progressive pump failure [14].

#### 2.2.2. Ischaemia

This paper focuses on monomorphic VT (i.e. VTs with uniform QRS morphology) in the post-MI population and not on arrhythmogenesis in the setting of acute ischaemia, most often leading to polymorphic VT (i.e. VTs with continuously changing QRS morphology). However, acute ischaemia might serve as a trigger for scar-related VT. Acute ischaemia causes depressed excitability of the ischaemic myocardium by potassium accumulation, calcium increase [50] and acidosis, leading to slowing of conduction and heterogeneous recovery of excitability [51], creating re-entry circuits in the border zone and giving rise to ventricular arrhythmias [52]. Therefore, persistent or intermittent ischaemia could serve as a trigger to scar-related, monomorphic VT.

Ischaemia can be examined by coronary angiography with fractional flow reserve measurement, as well as non-invasively by positron emission tomography [53] and single-photon emission tomography [54].

### 2.3. Modulators

#### 2.3.1. Markers of autonomic tone

Autonomic tone is the balance between the sympathetic (which triggers ‘fight or flight' reactions) and parasympathic (which triggers the ‘rest and digest' reactions) nervous system. The parasympathic nervous system is mediated by the vagal nerve and counteracts the sympathetic system. The proarrhythmic effects of increased sympathetic activity and reduced vagal activity are associated with increased SCD and VT [28]. Autonomic tone can be measured by several diagnostic tests. Baroreflex sensitivity (BRS) is evaluated as the change of the RR interval (i.e. beat length) to a blood pressure change [3]. Heart rate variability (HRV) describes beat-to-beat variation in heart rate. Heart rate turbulence (HRT) describes the heart rate response after a ventricular premature beat. A normal response is first acceleration followed by deceleration of the heart rate. HRT consists of the onset and the magnitude of the subsequent slowing in heart rate (HRT slope). Deceleration capacity (DC) provides an overall status of the autonomic, mostly vagal balance, by integrating all the regulatory processes that slow heart rate on a beat-to-beat basis [55]. Declines in HRV, HRT and BRS all indicate superfluous sympathetic tone.

There is a clear association between depressed HRV and arrhythmic events, as shown in the EMIAT trial [56]. HRV values are strongly associated with increased mortality and baseline heart rate in patients with reduced ejection fraction, possibly reflecting the sympathetic overdrive. The DINAMIT study [57] examined these patients with impaired cardiac autonomic function early after their MI. Interestingly, the overall survival did not improve by prophylactic ICD implantation. Therefore, markers of autonomic dysfunction possibly identified a subgroup at high risk for death due to progressive heart failure instead of VT [58]. In patients without a reduced LVEF, the ISAR-Risk trial [55] showed that the absence of severe autonomic failure generates a negative predictive value of 93.9%.

Combining autonomic factors probably improves risk prediction of cardiac arrhythmias. The REFINE study [39] showed that the combination of abnormal Holter (i.e. ambulatory ECG monitoring) MTWA plus HRT and LVEF under 50% resulted in the best identification of patients at risk for cardiac arrhythmias with an accompanying HR of 6.22. Current guidelines [46] advise against incorporation of these non-invasive autonomic dysfunction measures early after MI for risk stratification.

#### 2.3.2. Miscellaneous

Next to autonomic modulators, n-3 fish oil seems to play a preventative role in VT. The fish oil fatty acids electrically stabilize myocardial cells through modulation of fast voltage-dependent sodium channels and L-type calcium channels [59]. There is also a possibility that genetic predisposition might play a role. This has not been studied extensively, but a case study consisting of 10 patients indicates that the genetic architecture of patients with MI, might play a role in the development of VT. The described genetical variations are known genetic defects in channelopathies, but now described in patients with ischaemic cardiomyopathy [60].

Currently, from the pathophysiological origin of VT, only the triggers related to heart failure are incorporated in clinical risk stratification. Other possibly prognostic relevant information is hidden in the patient files and not effectively used. Data-driven and mechanistic modelling, Approaches 2 and 3 described in §§3 and 4, respectively, could help in providing better risk stratification by incorporating the available clinical diagnostics discussed in this section.

## 3. Approach 2: data-driven models

While there exists a wide variety of different computer models in cardiology, they can generally be divided into two categories: data-driven models and mechanistic models. Data-driven models aim to describe the data by discovering underlying relationships without any predefined knowledge. The most upcoming data-driven models are AI models. These models go beyond traditional statistical models in the sense that *a priori* knowledge about possible correlations between data need not be known. In contrast to mechanistic models, their parameters usually do not have a physical meaning and are automatically fitted to the accompanying data, also known as 'learning'. Once the parameters are learned, possible new complex relationships can be found beyond direct human interpretation. In this way, it can support physicians to integrate the excessive amount of available information and find possible new predictors of VT. In this section, we will provide an introduction of some data-driven techniques used for VT modelling as well as some examples of AI implementations for malignant VT and VT-ablation prediction.

### 3.1. Artificial intelligence models

The input for AI can be raw data or structured feature (i.e. property) vectors that mathematically represents the data. ML is a subdiscipline of AI which uses data to learn in a supervised or unsupervised manner. In supervised ML, the input has corresponding output labels such as the presence of disease or corresponding heart rate. The dataset is divided in training data, validation data and testing data, commonly 60%, 20% and 20% of the complete dataset, respectively, to overcome overfitting. The model uses the training dataset to learn the relationship between the input and the labelled output over many iterations. After training, the validation dataset is used to check the quality of the model and, if necessary, make according adjustment to the ML algorithm. This training and validation is usually repeated a few times to optimize the model. In the next phase, the testing phase, the performance of the trained model is evaluated with the unseen test data. The most critical component of model performance lies in feature engineering of the input data. The challenge of supervised learning is that a large amount of labelled data is necessary, which is a time-consuming task but also limited by regulatory and privacy-related restrictions. This also imposes inter-observer variability in correctly labelling output data. Unsupervised ML, on the other hand, quantifies natural patterns in input data and identifies relevant subgroups by finding underlying structure in the data. Therefore, this does not require labelling of output data. This technique still requires proper preparation of input data [61]. A mixed approach combining supervised and unsupervised learning is also possible [62].

DL is a subtype of ML and is a promising technique in cardiovascular research, since it can perform image and video processing and can handle complex raw input data without manual feature engineering. It is a multi-layer artificial neural network. Layers of nodes, neurons, are connected between different layers, synapses. The strengths of these connections are quantified by weights and the importance of a synapse by a bias. During the learning process, these weights and biases are adjusted. The most used DL exploits deep convolutional neural networks (CNNs). Each convolutional layer uses filters (feature maps) to detect data features (e.g. straight edges or curves), which becomes hierarchical in repeated layers. Recurrent neural networks are used to analyse a temporal sequence of data.

In supervised ML, it is difficult to predict what type of model will perform best, so model development and optimization are typically empirical. In traditional supervised ML, model development and optimization consist of selecting a learning algorithm, an optimal set of variables (also called a feature set), and tuning hyperparameters (main model settings) of the learning algorithm. In DL, model optimization includes testing different neural network architectures and tuning hyperparameters within the architectures. Some important hyperparameters in the DL training process are learning rate (step size per iteration), batch size (sample size per iteration) and epoch size (number of times the complete training dataset passes through the model). The hyperparameters of the architecture itself are number of nodes and layers, types of layers and activation functions, among others. Additionally, hyperparameters within the layers can be adjusted as well, such as filter sizes and number of filters in convolutional layers. For more extensive reviews of AI in cardiology, see the listed reviews [15–19] in the introduction and more specifically reviews [61–63] which focus on AI in arrhythmia.

### 3.2. Artificial intelligence applications

#### 3.2.1. Ventricular tachycardia identification

One of the main applications of AI in cardiology, focuses on the automatic delineation [64] and interpretation of ECGs and identification of specific conditions in ECGs [16]. Mjahad *et al.* [65] could identify VT on 12-lead ECGs using deep neural networks with an accuracy of 98.19%. With another deep neural network on single-lead ECGs, Hannun *et al.* [66] were even able to identify 12 rhythm abnormalities better than an average cardiologist, with an ROC of 0.97 and an average F_1_ score of 0.837 versus 0.780, where the F_1_ score is the harmonic mean of the precision and sensitivity. Though for VT specifically, the F_1_ score was lower (0.68 versus 0.77), this could be attributed to some debatable labelling of records as idioventricular rhythms. These rhythms mainly differ from VTs in having heart rates below 100 beats per minute. Relabelling seven idioventricular rhythms that contained periods of more than 100 beats per minute to VT, resulted in an F1 score of 0.82. Importantly, though individual reports have shown better performance than physicians, systemic reviews concluded they perform equivalently [67,68]. Nevertheless, the results of Mjahad *et al.* [65] and Hannun *et al.* [66] are encouraging examples of AI assisting in automatic identification of known conditions.

However, one of the true promises of AI is detecting subtle details in ECGs which may not be easily seen or interpreted by physicians. For instance, Attia *et al.* [69] could identify patients with ejection fraction less than or equal to 35% using a CNN on only ECG data instead of echo data with a ROC of 0.93, sensitivity of 86.3%, specificity of 85.7% and accuracy of 85.7% tested on almost 53 000 patients. Since ECG is cheaper and faster than echocardiograms, this would improve clinical efficiency. More examples of applications of ML on ECGs can be found in an abundance of review papers [70–72].

#### 3.2.2. Ventricular tachycardia prediction

Besides identification, data-driven models could potentially also enhance VT prediction, as shown by Okada *et al.* [73]. Using supervised ML, they classified the substrate of scarred myocardium into a complexity score which had a negative predictive value of 91% for VT as outcome. Although they were able to determine a low-risk subgroup, there was a poor positive predictive value, plausibly because VT does not only require the appropriate substrate, but also sufficient triggers that may fluctuate over time. Other mechanical properties besides scarring could also be used in analogy to the research of Balaban *et al*. [74], who used left ventricle three-dimensional (3D) shapes to predict VTs in dilated cardiomyopathy patients, and of Corral Acero *et al*. [75], who showed in a multi-centre cohort that 3D left ventricle shape and contraction metrics could predict major adverse cardiac events 1 year after MI.

Choi *et al.* [76] predicted one of the previously described triggers, heart failure, from data over a 12–18-month period from electronic health records, including demographics, clinical and laboratory values, medication prescriptions, alcohol and tobacco consumption and International Classification of Disease version 9 (ICD-9) codes among others. This shows the importance of including longitudinal data.

Tsuji *et al.* [77] were even able to predict ventricular fibrillation 5 min prior to the event with an accuracy of 82.5% using several intensive care unit measurements (ECGs, non-invasive continuous blood pressure and arterial oxygen saturation) which were preprocessed to indices related to modulators such as HRV and vagal tone intensity. In fact, Lee *et al.* [78] developed a neural network which could predict VTs 1 h before onset, which raises the question whether it is possible to identify this risk even earlier. This neural network used 14 parameters based on analysis of HRV and respiratory rate variability and had a sensitivity of 0.88, specificity of 0.82 and AUC of 0.93 using 52 VT cases.

#### 3.2.3. Ventricular tachycardia origin localization

The localization of the origin of VT plays an important role in VT ablation. Sapp *et al.* [79] developed a multiple linear regression method based on 12-lead ECGs to localize the origin of LV activation during catheter ablation by a population-based method with an accuracy of 12 ± 8 mm. With a patient-specific method the error reduced to about 5 mm using 11 pacing sites. In a prospective study, this accuracy was even within 3.5 mm [80]. Though DL algorithms for localization of the VT origins are still in their early developing stage, Gyawali *et al.* [81] developed a DL algorithm which improved the localization prediction with about 2 mm in comparison with three other baseline models (e.g. regression, standard sequential autoencoder and supervised CNN) while taking inter-subject variation into account.

Besides purely data-driven models, a mechanistic approach to model substrate, triggers and modulators could be helpful in understanding the pathophysiology of this arrhythmia.

## 4. Approach 3: mechanistic models

Mechanistic models define a model based on known physics, chemistry, biology and physiological mechanisms, which is commonly called *a priori* knowledge. These mechanisms are parametrically described with mathematical equations such as ordinary or partial differential equations. The parameters have a physical meaning and can thus be adapted to fit a patient's individual physiology. These models mimic the underlying phenomenon by first conceptualizing the domain (e.g. fluid, mechanical or electrical domains) and consequently add the known first principles and tune their associated parameters [82]. Once tuned, these models can be used to extrapolate forward in time, providing important information about the dynamics of the underlying physical system and show, for example, if a re-entry circuit is possible.

Three types of mechanistic models are used for VT modelling: electrical models, mechanical models and electromechanical models. They consist of different organization levels, from cell to organ level, and of different model complexity, from lumped parameter models (0D) to realistic 3D models.

### 4.1. Electrical models

Electrical models or electrophysiological models focus on the generation and propagation of action potentials. On a cellular level, these models describe the functioning of ion channels (K^+^, Na^+^ and Ca^2+^), buffers, ion pumps and transporters of a single myocyte in 0D. One of the most well-known models is the Hodgkin–Huxley model [83]. Other examples are among others the model of ten Tusscher *et al.* [84] and Mitchel & Schaeffer [85]. In addition, electrical models can incorporate Ca^2+^ homeostasis, β-adrenergic stimulation and pH regulation. Biophysical protein models have been used to study the effect of hypoxia, potassium accumulation and acidosis, which is caused by ischaemia, on individual proteins. These mechanistic models have been used in models on myocyte scale to predict how these proteins change phenotypes [86]. Additionally, several researchers have studied the relationship between calcium dynamics and action potential alternans which can result in MTWA, an electrical substrate for a VT (§2.1.2), using ionic models [87–89]. Elucidation of these examples can be found in this review paper [90]. These types of models have also been used to study the effect of mutations on channelopathies [91].

Modelling cell-to-cell communication for the entire myocardium using a biophysical approach is often computationally intractable. Therefore, action potential propagation on tissue level is often modelled with phenomenological models, like the bidomain [92] and monodomain models. These models can be used to describe the spatial propagation in 1D, 2D or 3D. They conceptually average the properties of the extracellular and intracellular space in muscle fibre groups. Bidomain models describe the intracellular and extracellular potential in the cardiac wall with two partial differential equations. These equations include the conductivities of the two domains, which are determined by the highly anisotropic (meaning properties have different values in different directions) fibre orientation. Monodomain models on the other hand assume equal anisotropic ratios, meaning the two conductivities are linked by a constant, to reduce the computational demand, even though it is physiologically less accurate. Since the membrane potentials result from ionic currents, the monodomain and bidomain equations on tissue level are coupled to a cellular model as described earlier. Other well-known models to approximate the action potential propagation are simple generic models like the Eikonal model [93], which replaces the ionic current formulation with mathematical equations of low computational costs.

In order to simulate the action potential propagation over the whole myocardium, a 3D geometry model has to be created. This geometry is often based on medical images, for example from MRI. Subsequently, it is meshed into elements resulting in a patient-specific 3D geometric model. Related methods such as isogeometric analyses (IGA) are also available for generating geometrical models. Next, a fibre orientation needs to be assigned for each element [94]. Since fibre orientation can hardly be measured in standard clinical practice, they are estimated typically using a rule-based approach based on histological and *ex vivo* diffusion tensor magnetic resonance imaging (DT-MRI) or an atlas-based approach which wraps new DT-MRI images to a cardiac fibre atlas. Subsequently, an appropriate electrical model can be attributed. Lastly, a finite-element, finite-volume or finite-difference method is used to approximate the solution of the model. A detailed description about electrical modelling can be found in the review of Clayton & Panfilov [95].

### 4.2. Mechanical models

Mechanical or mechano-biological models focus on describing the stress–strain relationships using equations of mechanics. These stress­–strain relationships are the underlying mechanisms describing pressure–volume relationships and cardiac wall deformations from which clinical measures like LVEF and cardiac output can be estimated. Simple 0D models such as the one-fibre model link parameters on fibre level (fibre stress and length) to parameters on a ventricular level (left ventricular pressure and volume). Even though these models only give a limited representation of material properties and geometry, they are of low computational costs and can provide basal insights in the relationship between local wall mechanics and global ventricular mechanics. The boundary conditions for these simple models and for complex 3D models are often lumped parameter models such as the Windkessel model [96]. These models use electrical elements like resistance, compliances and inertance to model vascular flow. However, the boundary conditions can also be estimated from measurements.

Besides 0D modelling, a vast variety of mechanical models in 1D, 2D or 3D exists, with 3D models being most prominent for VT modelling. For a full 3D mechanical model, first a geometry model, meshed into elements and including fibre orientations, has to be created as described previously. Next, appropriate material behaviour has to be assigned. For the myocardium, this consist of orthotropic (meaning in each individual perpendicular direction, properties are equal), hyper-elastic and nearly incompressible tissue with passive properties [22]. Consequently, one of the finite methods (finite-element, finite-volume or finite-difference) can be used to approximate the solution of the underlying physical laws (mass, momentum and energy balance) to calculate the stresses and strains in the myocardium.

### 4.3. Electromechanical models

A complete framework of the 3D electromechanical interplay, linking cellular electrophysiology, tissue mechanics and haemodynamics with clinically available data generally consist of the steps described above: create a 3D geometry, mesh this geometry into elements, assign fibre directions and appropriate properties, and solve the problem [20]. For an electromechanical model, the assigned properties are both electrophysiological as well as mechanical. In combination, these two properties form the electromechanical coupling, describing the cardiac wall deformation on organ level as a result of myocyte contraction on cellular level. Models which aim to create models solely based on non-invasive data can use patient-specific geometry but must use average human electrophysiology since this cannot be measured non-invasively. When of interest, mechano-electrical feedback or the cardiac conductance system can be incorporated as well. Additionally, research has been done on mechano-chemical interactions [97]. A more detailed description about 3D cardiac modelling can be found in Lopez-Perez *et al.* [20] and Trayanova & Rice [22].

This integrative biophysical approach allows mechanistic models to enhance available diagnostic data with physiological understanding. These models can be further extended to incorporate (patho-)physiological growth and remodelling processes. This creates biophysically enhanced clinical prediction models linking cellular electrophysiology, tissue mechanics and haemodynamics with clinically available data, which provides a promising tool for virtual patient-specific simulations. For more extensive reviews of mechanistic models in cardiology see the listed reviews [20–23] in the introduction and more specifically reviews [95,98,99], which focus on multi-scale cardiac modelling, and reviews [90,100], which focus on personalized models for arrhythmia prevention.

### 4.4. The virtual-heart arrhythmia risk predictor and virtual-heart arrhythmia ablation targeting models

Combining the knowledge of the anatomical and electrical substrate (see §2.1) into one integrative biophysical mechanistic model of the heart can provide vital information as shown by the state-of-the-art virtual-heart arrhythmia risk predictor (VARP) and virtual-heart arrhythmia ablation targeting (VAAT) models. The VARP model is a 3D electrical model created by Arevalo *et al.* [101] using 41 patient-specific ventricular models of post-infarct patients with LVEF less than 35% to predict infarct related arrhythmic SCD. The model generally follows the framework explained earlier: first reconstruction of the geometry based on MRI data, next fibre orientation estimation, and lastly electrophysiological properties assignment of the myocardium. In this model, the myocardium is classified in healthy, border zone and infarct core tissue with corresponding altered membrane kinetics. The infarcted area was modelled to have no conductivity, whereas the border zone was characterized by prolonged action potential and decreased peak amplitude, upstroke velocity and conductivity. In this cohort, VARP tests (i.e. tests to examine if an arrhythmia could be elicited with the model) were significantly related with arrhythmia, whereas LVEF and other risk predictors (infarct border zone volume, scar volume and LV mass) were not. The VARP model thus outperformed existing clinical metrics. Moreover, this method can also be used for post-infarct patients with preserved LVEF, who are still at risk for VTs. Deng *et al.* [102], made important steps towards this.

The main successor of this model is the patient-specific VAAT model of Prakosa *et al.* [103]. They determined the optimal personalized ablation lesions with their model prior to the clinical procedure. This has the potential to radically change VT ablation therapy providing precise and personal ablation targets and possibly even eradicate all re-entry circuits. The feasibility of such personalized electrophysiological VT computer models and the effect of different border zone modelling strategies have been investigated [104,105]. They showed the importance of accurately modelling electrophysical and structural properties of the border zone like conductivity, action potential duration and fibre orientation, as the presence or absence of VT inducibility depends on these properties. However, these properties are difficult or even impossible to measure non-invasively. Recently, even electromechanical models of VT including electromechanical feedback have been published that might further enhance these types of models [106,107].

## 5. Approach 4: the future — digital twin

Clinical studies are designed to guide physicians in choosing the correct treatment by comparing groups of patients with comparable cardiac pathologies. The individual patient, however, often does not comply with the average study population. There is need to tailor the treatment to the individual's pathology. As mentioned, data-driven as well as mechanistic models can play a vital role in this transition. However, they both have their limitations.

Data-driven models, such as AI, can discover new unknown relationships between input and desired outputs without the need of any predefined knowledge. A major limitation of these AI models, however, is their black box nature, which makes interpretability and trustworthiness challenging in clinical decision making. Additionally, a large amount of (labelled) data are needed and bias in these data is almost inevitable. Mechanistic models, on the other hand, can only be used if the underlying mechanisms are understood, which is in practice not often the case. Moreover, parameter tuning is ambiguous. Nonetheless, if the underlying mechanisms are known and computational effort and parameter tuning are feasible, mechanistic models are exceptionally powerful in improving our understanding, making predictions and testing different treatment options. Consequently, mechanistic models may be best if there is an acceptable understanding of the system, whereas data-driven models may be the appropriate choice if this understanding is lacking or the mechanistic models become too complex. However, the true merit lies in combining these two model approaches in a hybrid way, especially when applied in a digital twin concept.

### 5.1. The digital twin

A digital twin is a promising tool helping the physician in making the optimal decision for the individual patient. As mentioned earlier, a digital twin coherently and dynamically integrates clinical data acquired over time with mechanistic and data-driven models to create a virtual model of (part of) a patient [12]. A cardiac digital twin is thus essentially a real-time digital replica of the individual patient's heart, which is iteratively updated with up-to-date clinical data. Importantly, there is a two-way communication between the patient and the digital twin. Where the patient provides data to the digital twin, the digital twin will give suggestions and predictions to the patient. The digital twin uses both population and individual data to develop a model which optimally supports decision making. The digital twin could even be enhanced with lifestyle questionnaires or mobile sensors which can be worn at home, thereby provide real-time input. This can eventually even shift the clinical approach towards preventive care. Ultimately, this model can be applied for fast evaluation of personalized diagnosis, to forecast disease development, to guide treatment planning and to provide recommendations for preventive care.

**Figure 4.**
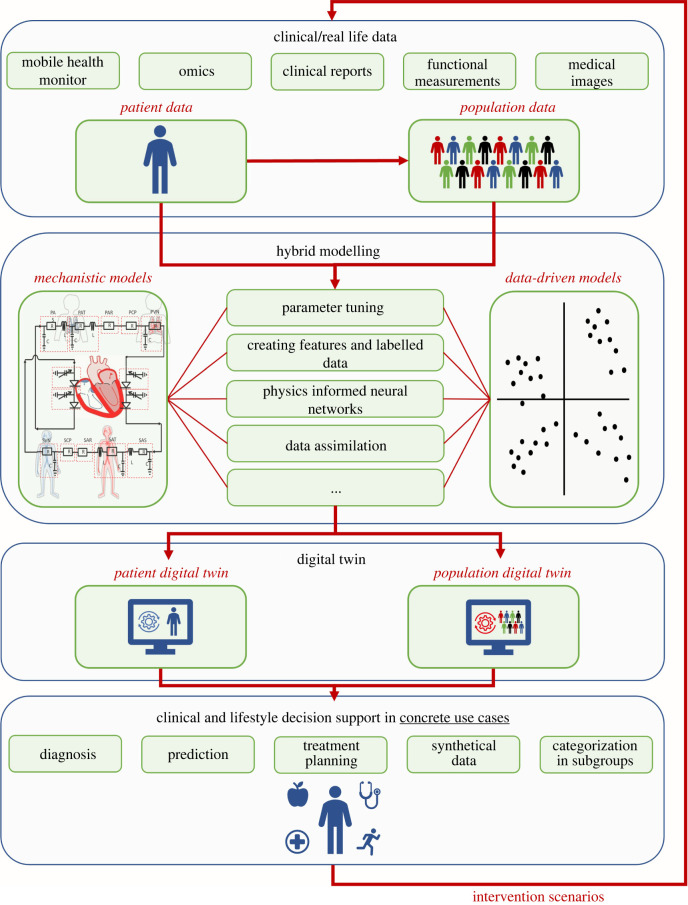
Detailed digital twin concept. Schematic visualization of the digital twin in which population and personal data are used to create a patient-specific digital twin based on a hybrid combination of mechanistic and data-driven models. This digital twin can help in clinical decision and lifestyle support for concrete use cases.

Another application of digital twins is the generation of synthetic patient data. These data can be used to replace missing data, better known as data imputation, or to create big datasets needed to train ML algorithms. Moreover, these virtual patients facilitate *in silico* clinical trials. These virtual trials can be used prior to an actual clinical trial to prevent failing trials, derive better inclusion and exclusion criteria, reduce time, perform trials when it would otherwise be unethical, and ultimately replace clinical trials [108]. A visualization of how a digital twin can be used is given in figure 4.

Although the term digital twin might seem to refer to an all-encompassing model, realistically it is more likely that multiple different digital twins will be created for concrete use cases, such as specific diseases and treatments. To create a digital twin, the most important components that need to be designed and constructed are the input measurements, the desired output and the model itself, which all need to fit within a clinical setting. As input measurements, we envision a combination of standard easy-to-perform measurements and detailed extensive data which will be added if diseases become more progressive.

As an illustrative example, the digital twin of a subject without any medical history will probably only require simple measurements like age, heart rate, blood pressure and body mass index, which could be complemented with other information like dietary and exercise patterns, all recorded by a mobile app. This digital twin of a healthy subject will mostly use these data to categorize the subject in a specific subject group according to population data. When the healthy subject changes to an unhealthier subject group due to, for example, ageing or changes in the measurements, the digital twin will recognize this and can give lifestyle recommendations. If the changes are, however, more serious, or an acute event has taken place such as a MI, the digital twin will acknowledge that more data are necessary and a more advanced digital twin is needed.

In this MI case, more extensive data need to be acquired, for example repeated ECG, echo, vital functions and laboratory measurements on top of the standard measurements. Additionally, details about medication, as well as the measurements obtained during, for example, the treatment of the MI itself, such as the angiography and ECGs, will be included. The digital twin of a patient who suffered MI will be more personalized, considering cardiac geometry, mechanics and electrophysiology while observing (small) changes with respect to the (yearly) acquired data. This digital twin will probably output the possibility of different cardiac events after infarction, like reinfarction, development of VT, or even death. When the model outputs high possibilities of VTs, the physician can decide to start testing this possibility with a digital twin for VTs.

This VT digital twin might require even more detailed data like MRI scans of the border zone and infarct core, Holter measurements and EPS for more accurate predictions. This model will therefore be highly patient-specific and can be used to test if VTs can be induced and if so, to test the use of ICD therapy versus (preventive) ablation. Moreover, ablation procedures could be first tested out on the digital twin itself and origins of VT could already be preselected.

After the procedure, a monitoring digital twin can be used, which will roughly be of the same complexity as the digital twin of the infarcted patient. After a while, when the patients are fully recovered, the first model for healthy subjects could be used again.

In summary, the digital twin will start simple, mostly relying on population models with measurements which can be measured at home and straightforward categorizations as output. However, with progressive disease development, the digital twin will become more personalized and complex and consequently needs more complex data input to provide predictions and test treatment options.

### 5.2. Hybrid modelling

To fully develop a digital twin, we envision hybrid modelling to be imperative in the model design. Hybrid modelling combines data-driven models and mechanistic models in such a way that both model types are fully exploited. Thus, part of a hybrid model is based on *a priori* knowledge and part of the model is inferred from data. Important to note is that measurements play a key role in hybrid models, as both data-driven as well as mechanistic models need certain data as input.

**Figure 5.**
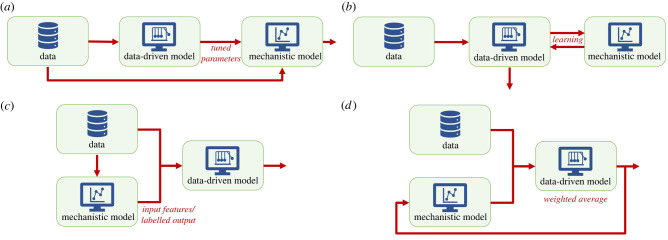
Hybrid modelling concepts. Schematic visualization of a few hybrid modelling examples. (*a*) A sequential method; (*b*) a physics informed neural network method; (*c*) a method where mechanistic models are used as feature input or as labelled output of the neural network; and (*d*) a data assimilation method.

There are many different configurations to assemble measurements, data-driven models and mechanistic models. One could imagine a sequential way (figure 5*a*) in which AI is, for example, used to interpret ECGs, and these outcomes are subsequentially used to tune the parameters describing action potential propagation in the electromechanical model. Another interesting sequential example could be the incorporation of risk factors, such as the clinically relevant modulators and triggers described in §2, in the mechanistic model by using AI. Currently, no physics law is known to describe the causal relationship between these factors and the occurrence of VT, and thus risk factors cannot be incorporated in the mechanistic models, even though evidence-based medicine has shown the relevance of risk factors. Discovering and consequently adding these types of relations is typically a data-based model problem. Similarly, AI could be used to predict how parameters of the mechanistic model change over a long period of time, as was done by Regazonni *et al.* [109]. This is important, as abnormal changes in these parameters could predispose disease development. Regazonni *et al.* [109] showed the effectiveness of their hybrid approach to classify and predict patient physiology with a 0D model of the blood circulation and a neural network. Importantly, they were able to create a neural network with parameters with physical meaning, which highly improves the interpretability.

Another up-and-coming hybrid methodology is physics informed neural networks (PINNs) [110]. PINNs incorporate physics law in the training process of the neural network (figure 5*b*). This requires less data and makes training more efficient, as these known laws regulate the learning process.

An alternative way to combine the two models is to use results of mechanistic models as feature input or as labelled output of the neural network (figure 5*c*). For example, Shade *et al.* [111] developed an ML method to predict which patients have a high risk of atrial fibrillation recurrence after pulmonary vein isolation using features from LGE-MRI and electrophysiological simulations of atrial fibrillation induction. The performance of the ML classifier remained similar when using only simulation features, but decreased when only image features were used, illustrating the importance of incorporation mechanistic models. Lozoya *et al.* [112] used a comparable feature augmentation for their ML algorithm to detect ablation targets from images alone. The introduction of features from personalized image-based electrophysiological models gave better performance of their algorithm than using image-based features alone. Another example of a similar approach is of Fossan *et al.* [113]. They used prior knowledge of a reduced-order model (i.e. a simplified model) as input features to learn the discrepancy between this reduced-order model and a 3D model, which enhanced the predicted fractional flow reserve over pure ML models. All three previously discussed studies also showed that smaller datasets can be used when mechanistic models are included in the AI algorithms.

Other data-driven models than AI can also be used for hybrid modelling. For example, with data assimilation (figure 5*d*) a mechanistic model is used to make a prediction, and this prediction is subsequently adjusted to the available data using a Bayesian approach (a data-driven approach based on probabilities) to create a weighted average of the mechanistic model and the data. This in theory creates better results as data are often hampered by noise which can be diminished by incorporating known physics, while at the same time the data can compensate for unknown underlying physics. Of course, many more different hybrid configurations can be thought of and choosing the appropriate one is far from trivial.

### 5.3. Open challenges and gains

The open technical challenges are thus what data and which models should be used and how to integrate them seamlessly. Other difficulties lie in the low quality and quantity of medical data and the lack of verification and validation with well-designed longitudinal studies for data-driven and mechanistic models. Nonetheless, we do not have to start from scratch. Advanced components for this twin model already exist such as the population-derived automated VT exit localization of Sapp *et al.* [79] (data-driven model, §3) and the VAAT model [103] (mechanistic model, §4). A recent retrospective feasibility study showed that VT exit site prediction of both methods are concordant, and they thereupon advocated that a combination of the two would yield improved comprehensive ablation strategies [114]. Components like this will gradually be tailored and extended, eventually creating digital twins. Besides the technical challenges, there are additional practical challenges, like privacy, legal ramifications, accountability, the complication of data sharing across institutions, and the integration of these models into the complex existing clinical workflow [115]. To tackle these challenges, a close collaboration between clinical, scientific, industrial and regulatory departments is needed. It is of great importance that especially physicians have a key role in the design, implementation and interpretation of these models. In this way, a digital twin can be created in which evidence-based medicine, data-driven models and mechanistic models form a synergy which supports the physician in decision making and has the potential to truly transform the current clinical practice from population based to personal care.

## 6. Conclusion

Currently, risk stratification in patients with ischaemic cardiomyopathy is solely based on LVEF and symptoms. This was concluded from big cohort studies but is in daily practice not always applicable to the individual patient. Incorporation of patient-specific information on the substrate, triggers and modulators causing VT would greatly benefit the treatment of the individual patient. To optimize this prediction, there needs to be a hybrid integration of patient-specific data using data-driven and mechanistic modelling of the individual anatomical, mechanical and electrophysiological characteristics. The creation of this digital twin might benefit the individual treatment of the patient.

## Data Availability

This article has no additional data.
